# A Global Mutational Profile of SARS-CoV-2: A Systematic Review and Meta-Analysis of 368,316 COVID-19 Patients

**DOI:** 10.3390/life11111224

**Published:** 2021-11-11

**Authors:** Wardah Yusof, Ahmad Adebayo Irekeola, Yusuf Wada, Engku Nur Syafirah Engku Abd Rahman, Naveed Ahmed, Nurfadhlina Musa, Muhammad Fazli Khalid, Zaidah Abdul Rahman, Rosline Hassan, Nik Yusnoraini Yusof, Chan Yean Yean

**Affiliations:** 1 Department of Medical Microbiology and Parasitology, School of Medical Sciences, Universiti Sains Malaysia, Kubang Kerian 16150, Kelantan, Malaysia; wardahyusof@usm.my (W.Y.); irekeola@student.usm.my (A.A.I.); wadayusuf@student.usm.my (Y.W.); engkunursyafirah@student.usm.my (E.N.S.E.A.R.); naveed.malik@student.usm.my (N.A.); drzaidah@usm.my (Z.A.R.); 2 Microbiology Unit, Department of Biological Sciences, College of Natural and Applied Sciences, Summit University, Offa 250101, Kwara State, Nigeria; 3 Department of Zoology, Faculty of Life Sciences, Ahmadu Bello University, Zaria 810107, Kaduna State, Nigeria; 4 Human Genome Centre, School of Medical Sciences, Universiti Sains Malaysia, Kubang Kerian 16150, Kelantan, Malaysia; fadhlina@usm.my; 5 Institute for Research in Molecular Medicine (INFORMM), Universiti Sains Malaysia, Kubang Kerian 16150, Kelantan, Malaysia; fazlikhalid@usm.my (M.F.K.); nikyus@usm.my (N.Y.Y.); 6 Hospital Universiti Sains Malaysia, Universiti Sains Malaysia, Kubang Kerian 16150, Kelantan, Malaysia; 7 Department of Hematology, School of Medical Sciences, Universiti Sains Malaysia, Kubang Kerian 16150, Kelantan, Malaysia; roslin@usm.my

**Keywords:** COVID-19, SARS-CoV-2, mutation, mutational profile

## Abstract

Since its first detection in December 2019, more than 232 million cases of COVID-19, including 4.7 million deaths, have been reported by the WHO. The SARS-CoV-2 viral genomes have evolved rapidly worldwide, causing the emergence of new variants. This systematic review and meta-analysis was conducted to provide a global mutational profile of SARS-CoV-2 from December 2019 to October 2020. The review was conducted according to the Preferred Reporting Items for Systematic Reviews and Meta-analysis (PRISMA), and a study protocol was lodged with PROSPERO. Data from 62 eligible studies involving 368,316 SARS-CoV-2 genomes were analyzed. The mutational data analyzed showed most studies detected mutations in the Spike protein (*n* = 50), Nucleocapsid phosphoprotein (*n* = 34), ORF1ab gene (*n* = 29), 5′-UTR (*n* = 28) and ORF3a (*n* = 25). Under the random-effects model, pooled prevalence of SARS-CoV-2 variants was estimated at 95.1% (95% CI; 93.3–96.4%; *I*^2^ = 98.952%; *p* = 0.000) while subgroup meta-analysis by country showed majority of the studies were conducted ‘Worldwide’ (*n* = 10), followed by ‘Multiple countries’ (*n* = 6) and the USA (*n* = 5). The estimated prevalence indicated a need to continuously monitor the prevalence of new mutations due to their potential influence on disease severity, transmissibility and vaccine effectiveness.

## 1. Introduction

Coronavirus Disease-19 (COVID-19) is caused by Severe Acquired Respiratory Syndrome Coronavirus-2 (SARS-CoV-2) [1,2]. Since the SARS-CoV-2 epidemic first reported in Wuhan, China, the clinical features of COVID-19 have evolved, moving from clinically apparent pulmonary or flu-like symptoms to subclinical or even silent infections. The COVID-19 infection could frequently involve an asymptomatic or paucisymptomatic framework, leading to a spread in the general population [3]. COVID-19 produces respiratory distress with mild to severe symptoms, and it is fatal in individuals with a chronic disease or a compromised immune system [4]. Various clinical outcomes in COVID-19 patients have also been documented throughout several other regions across the world. As of 29 September 2021, according to the World Health Organization (WHO), the SARS-CoV-2 pandemic has infected over 232,075,351 individuals across the world, resulting in 4,752,988 fatalities and significant disruptions to regular activities and national economies.

The international scientific community continuously characterized the pathophysiological features of COVID-19, developed diagnostic tools, evaluated immune responses, and identified risk factors for severe illness courses. SARS-CoV-2 clustered outbreaks and super spreading episodes provide a unique challenge to pandemic control [5]. However, the basic characteristics of SARS-CoV-2 genome evolution and transmission dynamics within the human population are still unknown [6]. COVID-19 infection demonstrated related inflammatory state of the upper airway mucosa and olfactory neurotoxic damage. However, to date, a reliable method in the evaluation of the nasal health of post-infection patients is not clear [7].

SARS-CoV-2 genomic sequencing from several geographical regions has recently revealed that the virus quickly changes by accumulating mutations in its genome. It has been proposed that new SARS-CoV-2 variants may adapt better to new geographical locations, making them more potent than the virus that discovered in Wuhan, China.

All viruses’ genomes gain mutations over time. However, various variables, including the mutation rate and the effects of mutation on viral dynamics within and between individual hosts, influence the rate of mutation accumulation and its repercussions for transmission and illness in the host population [8]. The combination of these variables determines the development and transmission of viral variations and the evolution of pandemics. Detection of mutations spread worldwide is essential for a better understanding of the viral evolution, bio-pathology and transmission since RNA virus genomes are highly susceptible to mutation [9].

## 2. Materials and Methods

### 2.1. Study Design and Protocol

The Preferred Reporting Items for Systematic Reviews and Meta-Analysis Protocol (PRISMA-P 2015) guidelines [10] were used as this study’s checklist. The study population included individuals with SARS-CoV-2 infection with the main out-come being mutations in the SARS-CoV-2. Reference was made to the Wuhan strain as a comparator A Prospero protocol (No CRD42021229620) was lodged for this study.

### 2.2. Literature Review

The PROSPERO database and Database of Abstracts of Reviews of Effects (DARE) (http://www.library.UCSF.edu; accessed on 10 January 2021) were searched to ensure no other meta-analysis on the impact of the mutational profile of SARS-CoV-2 on transmissibility and disease severity exists or is ongoing. The literature search was performed using international databases PubMed, Scopus, Science Direct and Google Scholar using the search terms listed in Table S2. Two authors carried out the database search to minimize bias.

### 2.3. Inclusion and Exclusion Criteria for Studies

Inclusion criteria: (1) Studies reporting on human COVID-19, (2) Studies reporting on SARS-CoV-2 mutations, (3) Studies reporting on SARS-CoV-2 mutations and their association with superspreading events, transmissibility and severity of illness in COVID-19 patients. Exclusion criteria include reviews papers, animal studies, protein characterization studies, studies on environmental sampling, and media reports.

### 2.4. Quality Assessment

The methodological quality of the included studies was assessed independently by two authors using the Joanna Briggs Institute (JBI) critical appraisal checklist for prevalence data [11]. A score of ‘1’ for “yes” and ‘0’ for other parameters was assigned to attain a total quality score ranging from ‘0’ to ‘9’. Studies with an overall score of ‘7’–‘9’ were considered sufficient quality (Table S3).

### 2.5. Data Extraction

Two independent authors performed the data extraction by using standardized forms, which included manuscript title, authors, journal, publication year, countries of study, period of study, number of participants, number of mutated cases, regions of mutations, types of mutations, mutations, viral load, symptoms, severity (mild, moderate, severe, fatal), sample types (nasopharyngeal swab, bronchoalveolar lavage), viral shedding, co-morbidity, mutation detection method, the database used (data downloaded), database accessed and transmissibility.

Studies that analyzed genetic mutations from more than one country were categorized as “multiple countries” rather than the individual countries included. When mutational data from different countries and regions were analyzed as a whole, instead of by specific countries, they were characterized as ‘worldwide’, and the data were extracted and analyzed in that form to avoid confusion. For regions of mutations labelled as ORF1a, ORF1b, nsp1-14, 3C-like proteinase, RNA-dependent RNA polymerase (RdRp), helicase, 3′-to-5′ exonuclease, endoRNAse, 2′-O-ribose methyltransferase, or leader protein, they were characterized as ‘ORF1ab’ to simplify analysis. Where more than one article reported mutational data from the same group of sample, record, or patient cohort, only one was counted and selected.

### 2.6. Data Synthesis and Analysis

Data analysis was conducted using Comprehensive Meta-analysis Software (CMA) (Version 2.0) (https://www.meta-analysis.com/; accessed on 25 July 2021). The pooled prevalence of SARS-CoV-2 variants was calculated and subgroup analysis was done according to country. A random-effect model using the DerSimonian-Laird method of the meta-analysis was employed to determine the pooled estimates of the reported SARS-CoV-2 variants and subtype proportions. A forest plot was subsequently generated to visually summarize details of the individual studies alongside the estimated common effect and degree of heterogeneity. Publication bias was examined using funnel plots (visual aid for detecting bias) and Egger’s regression test. Cochran’s Q test evaluated the heterogeneities (i.e., variation in study outcomes between studies) of study-level estimates and quantified using *I*^2^ statistics. *I*^2^ values of 25%, 50%, and 75% were considered low, moderate, and high heterogeneity, respectively [12].

Subgroup meta-analysis was used to analyze sources of heterogeneity. A sensitivity test was conducted using the leave-one-out analysis. *p*-value of <0.001 was considered to be statistically significant for all tests.

## 3. Results

### 3.1. Search Result and Eligible Studies

**Figure 1:**
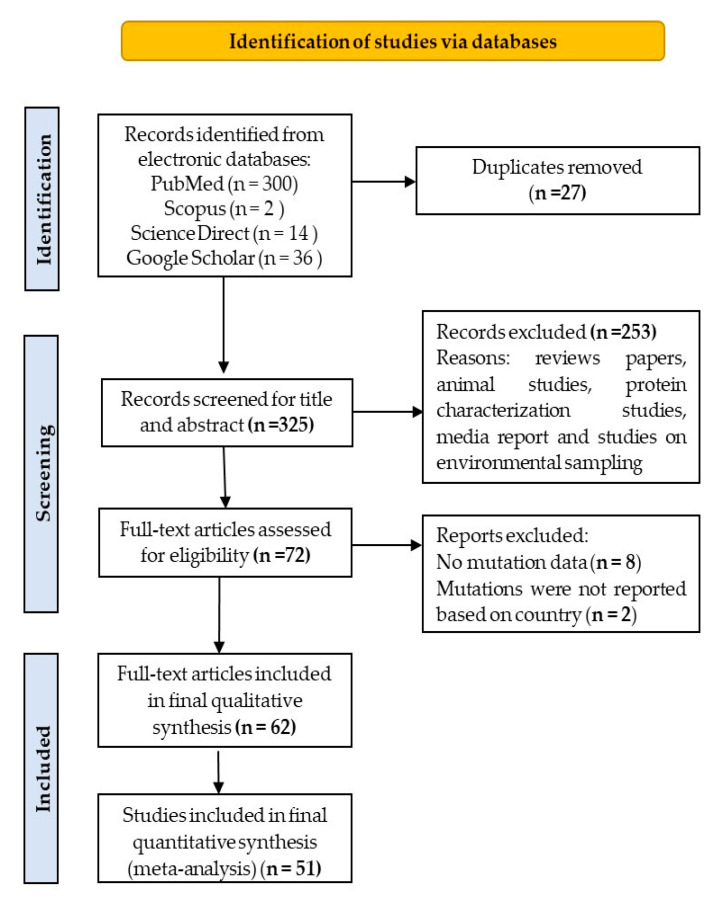
PRISMA flow diagram for the selection og eligible articles included in the study.

The complete literature search process is displayed in Figure 1. The search strategy initially found 352 articles, after which 325 were left after duplicates removal. Two hundred and fifty-three articles were excluded based on the exclusion criteria. The full-text of 72 articles were assessed for eligibility, and ten were excluded for lack of mutations data or mutations data were not countries-specified. A total of 62 articles were included in the final qualitative synthesis, and finally, 51 articles published between December 2019 and October 2020 were included in the final quantitative synthesis (meta-analysis).

### 3.2. Characteristics of the Eligible Studies

All the eligible studies included in the meta-analyses were of high methodological quality. From 62 studies included from December 2019 to October 2020 (Table 1) [2,13,14,15,16,17,18,19,20,21,22,23,24,25,26,27,28,29,30,31,32,33,34,35,36,37,38,39,40,41,42,43,44,45,46,47,48,49,50,51,52,53,54,55,56,57,58,59,60,61,62,63,64,65,66,67,68,69,70,71,72,73], the highest numbers were from Worldwide (*n* = 10), multiple countries (*n* = 6) and the USA (*n* = 5). The 368,316 samples and genomic data analyzed in the studies were detected by quantitative Reverse Transcriptase (qRT-PCR) or DNA sequencing (Sanger, Next-generation, Whole-genome or Nanopore sequencing).

**Figure 2:**
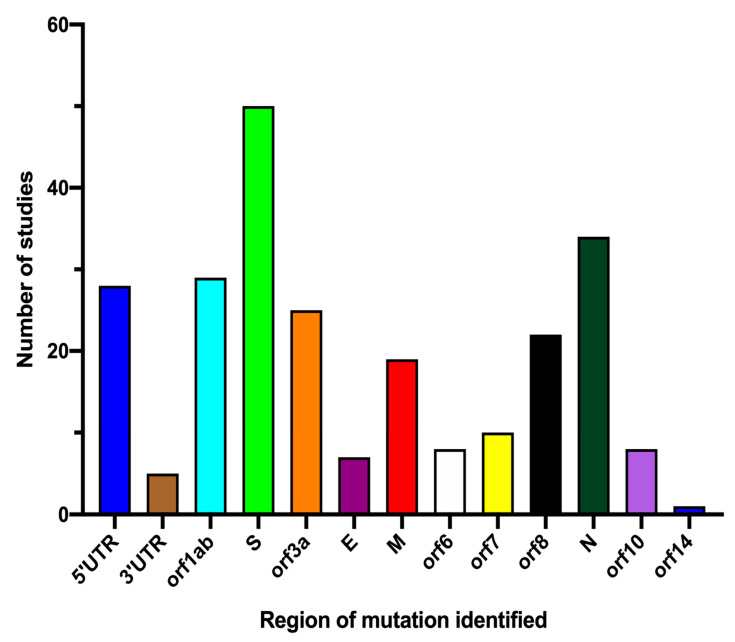
Reported regions of SARS-CoV-2 mutation. Data presented is based on the identification of mutation in any of the highlighted genomic regions from the included studies that presented data on region of mutation (*n* = 62). Some studies reported more than one region.

Genomic data from the studies included covered all regions of SAR-CoV-2 (Figure 2). From the mutational data analysed, the studies detected mutations in the Spike (S) protein (*n* = 50), Nucleocapsid (N) phosphoprotein (*n* = 34), Open Reading Frame (ORF) 1ab gene (*n* = 29), 5′-Untranslated region (UTR) (*n* = 28), ORF3a (*n* = 25), Membrane (M) glycoprotein (*n* = 19), ORF7 (*n* = 10), ORF6 (*n* = 8), ORF8 (*n* = 8), ORF10 (*n* = 8), Envelope (E) protein (*n* = 7), 3′ UTR (*n* = 5) and ORF14 (*n* = 1). The synonymous and missense mutations detected, mostly based on countries and region of mutations, are listed in Table S1.

### 3.3. The Pooled Prevalence of SARS-CoV-2 Variants

**Figure 3:**
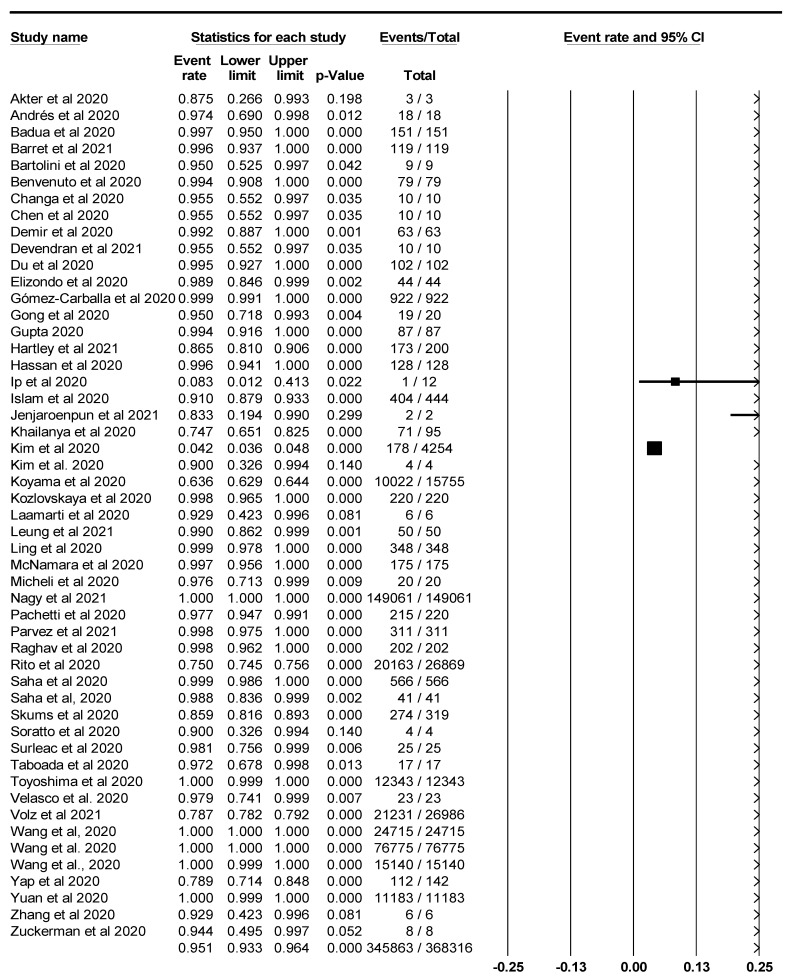
Forest plot showing the pooled prevalence of SARS-CoV-2 variants.

**Figure 4:**
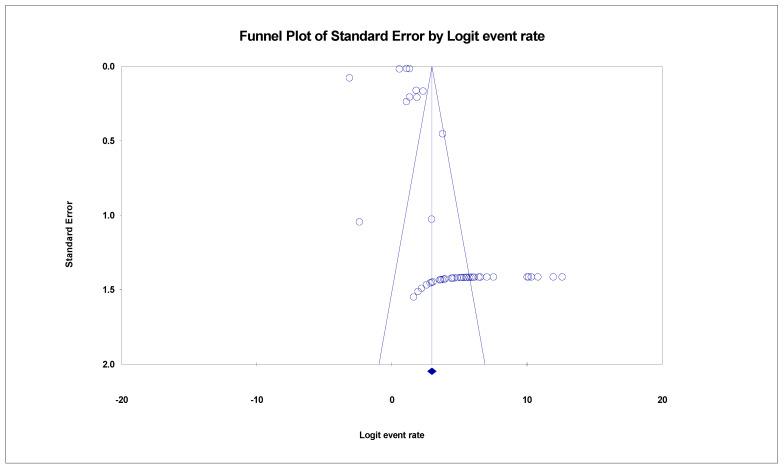
Funnel plot showing publication bias in studies reporting the prevalence of SARS-CoV-2 variants.

**Figure 5:**
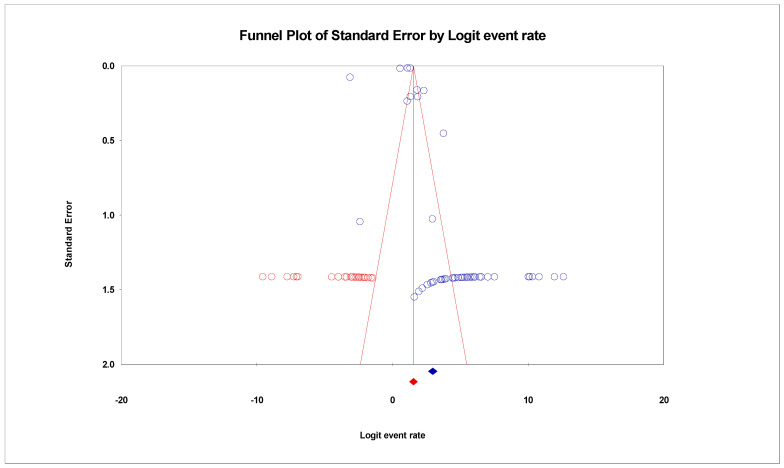
Funnel plot of the prevalence of SARS-CoV-2 variants showing 22 added studies (in Red) in the Trim-and- Fill method.

The pooled prevalence of SARS-CoV-2 variants was estimated at 95.1% (95% CI; 93.3–96.4%; *I*^2^ = 98.952%; *p* = 0.000) (Figure 3). Random-effects meta-analyses were carried out. Between-study variability was high (*t*^2^ = 0.515; heterogeneity *I*^2^ = 98.952% with heterogeneity chi-square (Q) = 4772.621, degrees of freedom (df) = 50, and *p* = 0.000). Moreover, publication bias was observed, as shown in the asymmetrical funnel plot (Figure 4). Using the Trim and Fill method and because the random-effects model was utilized, 22 missing studies were imputed to the left side of the mean effect (Figure 5), resulting in a point estimate of 82.5% (95% CI; 77.6–86.4). In addition to the funnel plots, Egger’s test was used to confirm the extent of bias (*t*-value = 1.447; *p* = 0.07717).

### 3.4. Subgroup Meta-Analysis

**Figure 6:**
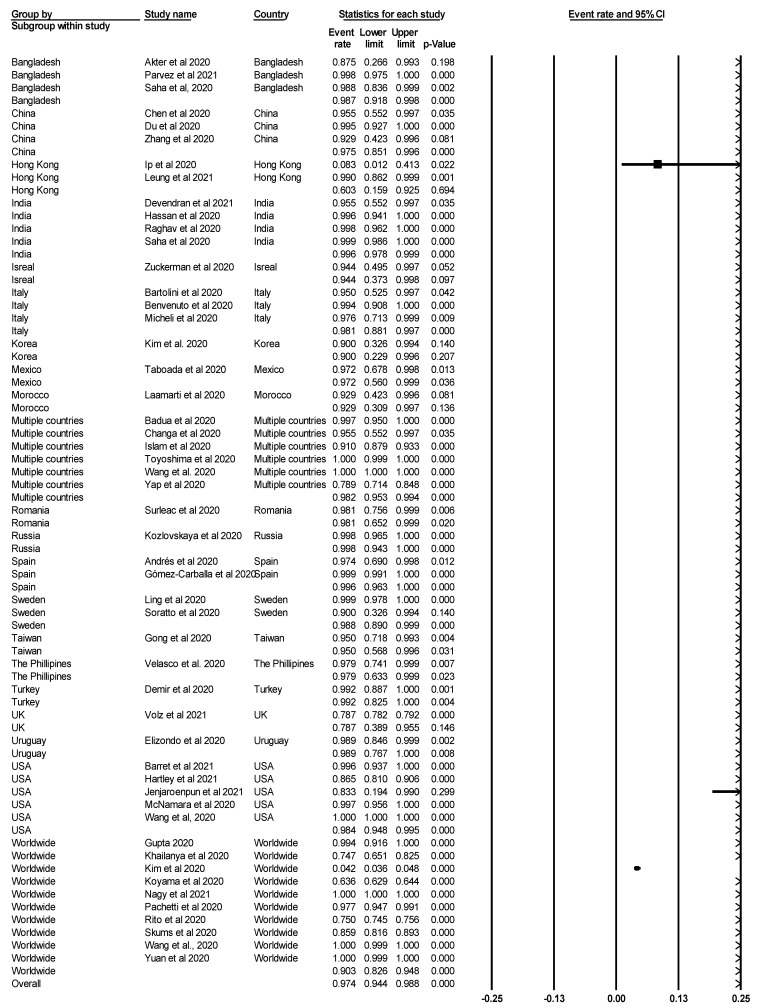
Forest plot showing the subgroup meta-analysis by country.

The result of subgroup meta-analysis by country showed that the majority of the studies were conducted Worldwide (*n* = 10), followed by studies carried out in Multiple countries (*n* = 6) and the USA (*n* = 5). Interestingly, China with three studies had heterogeneity (*I*^2^) of 5.356 and prevalence of 97.5% (CI = 85.1–99.6%), while Italy, with the same number of studies, had heterogeneity of 0.000 and prevalence of 98.1% (CI = 88.2–99.7%). Heterogeneity was highest among studies conducted Worldwide (*I*^2^= 99.747%), which was also trailed by six studies conducted in Multiple countries (*I*^2^= 95.168%) (Table 2). The forest plot is shown in Figure 6.

### 3.5. Meta-Regression

**Figure 7:**
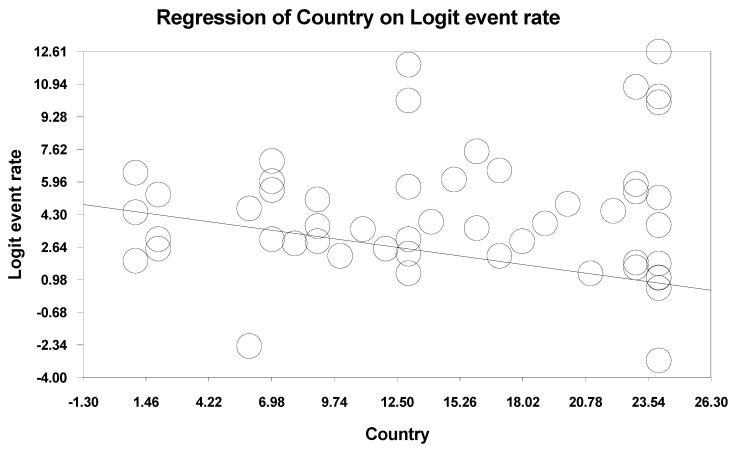
A scattered plot of Country Meta-regression.

Meta-regression was done for the single variable country. Method of moments was used as the computational option, and a scattered plot (Figure 7) was plotted. *p*-value of ‘0.000′ was obtained for ‘Country’, indicating the heterogeneity observed in this study, aside from chance, could also be contributed by country.

## 4. Discussion

With the high infection numbers worldwide, the SARS-CoV-2 virus has evolved, developed mutations and given rise to new genetic variations with increased infectivity and transmissibility. Efforts are currently being undertaken to characterize the virus and its genomic variability molecularly. Viral mutations and variants around the globe are routinely monitored through sequence-based surveillance, epidemiological analysis and laboratory studies.

This study has examined the mutational profile of SARS-CoV-2 between December 2019 to October 2020 from 62 studies of different continents. The pooled prevalence of SARS-CoV-2 variants in COVID-19 patients’ samples estimated by the random-effect model was 95.1%. Upon using the Trim and Fill method to adjust for potential bias, the estimate for the prevalence of the variants was still very high at 82.5%.

The analysis showed that between-study variability was high (*I*^2^ = 98.95%). The sub-group meta-analysis showed that the high heterogeneity was contributed by countries such ‘Worldwide’ (*I*^2^ = 99.7%), ‘Multiple Countries’ (*I*^2^ = 95.2%), Hong Kong (*I*^2^ = 93.7%) and USA (*I*^2^ = 92.3%). Only two countries, Italy (*I*^2^ = 0%) and China (*I*^2^ = 5.4%) showed a low heterogeneity score. The different methods used to detect the mutations may contribute to the high heterogeneity, especially in the ‘Worldwide’ and ‘Multiple Countries’. The high heterogeneity could also be attributed to the different regions of the SARS-CoV-2 gene analyzed (Spike protein, ORF1ab, Nucleocapsid polyprotein, ect.) and the type of samples used in the studies. Most of the studies, especially those referred to as ‘Worldwide’ and ‘Multiple Countries’, analyzed patients’ genomic data downloaded from GISAID’s database.

Most of the reported mutations were located at the Spike gene region, followed by the Nucleocapsid gene and ORF1ab gene. The high number of studies reporting on the Spike gene region might be due to its importance in the pathogenicity and transmissibility of the SARS-CoV-2 virus. The Spike (S) gene has two domains: S1 and S2. The S1 domain mediates receptor binding while S2 mediates downstream membrane fusion [74]. The S1 receptor-binding domain (RBD) shows a high affinity for the human ACE2 receptor in the lungs’ alveolar type 2 (AT2) cells. Once the virus is attached to the host cell receptor, cleavage occurs between subunits S1 and S2. The subunit S2 will drive the viral and cellular membranes to fuse. The S1 recognizes and binds to the ACE2 receptor, whereas S2 directly facilitates entry into the host cell, making S1 and S2 crucial for infection [14]. 

Data extracted from publications included in this study showed that a 23403A>G mutation in the S gene, which produced a missense mutation of D614G in the Spike protein, was recorded in 43 out of 62 studies. The D614G substitution is usually linked to three other mutations: a 241C>to-T mutation in the 5′-UTR region, a synonymous 3037C>T mutation, and a non-synonymous 14408C>T mutation at the RNA-dependent RNA polymerase (RdRP) known as P323L or P4715L at ORF1ab gene. 

Our data showed that D614G was detected in the European region from middle to late February 2020 [51]. By early March, it had spread rapidly to the United States (US) [51] and the South American region [62]. In east Asia, the D614G variant was found in Thailand from a sample diagnosed with COVID-19 in early March 2020 [69]. While in China, the variant was detected in samples collected from January to April 2020 [25]. By June 2020, D614G was found in every sample sequenced worldwide [75]. 

The mutation appeared to arise independently to simultaneously sweep across multiple geographic regions, suggestive of natural selection and an adaptive benefit of D614G. However, subsequent sequencing efforts identified the D614G mutation in viruses in several Chinese provinces in late January (first D614G in China: hCoV-19/Zhejiang/HZ103/2020; 24 January 2020), raising the possibility that global spreading of this mutation may result from chance founder events. Viruses carrying 614G mutation could initiate most early transmission events in multiple locations, demonstrating that D614G mutation was not adaptive, despite in vitro data showing its effects on receptor binding [76].

A study of more than 25,000 sequences of the UK population found that viruses bearing 614G mutation are associated with higher viral load and younger age of patients. It appeared to spread faster and seed larger phylogenetic clusters than viruses with 614D; however, no association was found between the presence of the Spike 614G with clinical severity and COVID-19 mortality [65].

In this study, few limitations were identified, including the inability to assess the impact of the identified mutations on patients’ viral loads, severity of the disease, and its transmissibility, due to the lack of reported data from the included studies. An understanding of the impact of the mutations on these variables would be invaluable. Furthermore, most of the studies downloaded only viral genomic data extracted from COVID-19 patients from NCBI and GSAID websites, thus limiting our access to the patients’ demographic information such as sex and age; and clinical data such as viral loads symptoms, co-morbidities and disease severity. The scarcity of the required data also limited the subgroup meta-analyses that could be conducted.

## 5. Conclusions

In this study, a systematic review and meta-analysis of studies were conducted to report the global prevalence of SARS-CoV-2 variants, estimated at 95.1%. Although a high heterogeneity was observed, we believe the estimate provides a good indication of the prevalence of SARS-CoV-2 variants worldwide from December 2019 to October 2020. With the fast evolution of the SARS-CoV-2 virus, there is a need to continuously monitor the prevalence of new mutations due to their potential influence on disease severity, transmissibility, resistance to antiviral drugs and vaccine effectiveness.

## Figures and Tables

**Table 1:** Major characteristics of the included studies.

No	Study ID (Ref)	Country of Study	Period of Study	No. of Participant	No. of Mutated Cases	Mutation Detection Method	Regions of Mutation
1	Akter et al., 2020 [13]	Bangladesh	May–June 2020	3	3	Whole-genome sequencing	ORF1ab, N and S gene
2	Andrés et al., 2020 [14]	Spain	March 2020	18	18	Deep sequencing of S gene	S gene
3	Badua et al., 2020 [15]	Multiple countries	January–May 2020	151	151	NGS	ORF1ab, ORF8, ORF3a, 5′UTR, 3′UTR, ORF6, ORF7a, ORF10, S, E, M and N gene.
4	Barret et al., 2020 [16]	USA	December 2019–May 2020	119	119	NGS	5′UTR, ORF1ab, S gene
5	Bartolini et al., 2020 [17]	Italy	February–March 2020	9	9	NGS (SARS-CoV-2 panel)	ORF1ab, UTR, S, N and M gene,
6	Becerra-Flores 2020 [18]	Worldwide	March–April 2020	NR	NR	NGS	S gene
7	Benvenuto et al., 2020 [19]	Italy	January–April 2020	79	79	NGS	S and N gene
8	Chang et al., 2020 [20]	Multiple countries	NR	10	10	NGS	ORF1ab, ORF8, S and E gene
9	Chen et al., 2020 [21]	China	January–February 2020	10	10	qRT-PCR on ORF1ab and N gene; RNA sequencing	ORF1ab, ORF3a, ORF8, ORF10, S and N gene
10	Cusi et al., 2020 [22]	Italy	March 2020	1	1	Direct RNA and amplicon sequencing	S gene
11	Demİr et al., 2020 [23]	Turkey	March–May 2020	63	63	NGS	ORF1ab, ORF3a, 3′UTR, 5′UTR, S, N and M gene
12	Devendran et al., 2021 [24]	India	as of April 2020	10	10	NGS/WGS	ORF1ab, ORF8, S and N gene
13	Du et al., 2020 [25]	China	January–April 2020	102	102	qRT-PCR, meta-transcriptomic sequencing	5′UTR, ORF1ab, S, ORF3a, ORF8, N gene
14	Elizondo et al., 2020 [26]	Uruguay	March–May 2020	44	44	qRT-PCR, NGS	ORF8, ORF3a, ORF1ab
15	Eskier et al., 2020 [27]	USA and UK	January–March 2020	11,701	11,701	NGS	Whole genome
16	Gómez-Carballa et al., 2020 [28]	Spain	as of June 2020	922	922	NGS	Whole genome
17	Gong et al., 2020 [29]	Taiwan	January–March 2020	20	19	RT-PCR & WGS	ORF1ab, ORF8, ORF3a, S gene, N gene
18	Gupta 2020 [30]	Worldwide	January–April 2020	87	87	NGS	ORF1ab, ORF3a, ORF7a, ORF8, N, S and M gene
19	Hartley et al., 2021 [31]	USA	March–June 2020	200	173	NGS	ORF1ab, S gene
20	Hassan et al., 2020 [32]	India	as of May 2020	128	128	NGS	ORF1, ORF3a, ORF8, ORF7a, S, M and N gene
21	Yang et al., 2020 [33]	Worldwide	December 2019–June 2020	46,414	46,414	NGS	Whole genome
22	Ip et al., 2020 [34]	Hong Kong	January–March 2020	12	1	Sanger sequencing, Nanopore and Illumina sequencing	S gene
23	Islam et al., 2020 [35]	Multiple countries	as of May 2020	444	404	NGS	ORF1ab, N, E, M, S
24	Jacob et al., 2020 [36]	India	until June 2020	>600	NR	NGS/WGS	S gene
25	Jary et al., 2021 [37]	France	January–February 2020	1	1	NGS	ORF3a, ORF7a, ORF6, ORF7b, ORF8, ORF10, N, M and E gene
26	Jenjaroenpun et al., 2021 [38]	USA	July 2020	2	2	Oxford Nanopore Technologies (ONT) MinION sequencing technology	ORF1ab, ORF3a, ORF14 and S gene
27	Khailany et al., 2020 [39]	Worldwide	December 2019–April 2020	95	71	NGS/WGS	ORF1ab, ORF8, ORF3a, ORF10, S, N and M gene
28	Kim et al., 2020 [40]	Worldwide	NR	178	178	NGS	ORF1ab, ORF3, ORF6, ORF7a, ORF7b, ORF8, ORF10 S, M, E and N gene
29	Kim et al., 2020 [41]	Korea	NR	4	4	qRT-PCR and Sanger sequencing	S gene
30	Koyama et al., 2020 [42]	Worldwide	February–May 2020	15,755	10,022	NGS	Whole genome
31	Kozlovskaya et al., 2020 [43]	Russia	March–April 2020	220	220	NGS	ORF1ab, S and N gene
32	Kumar et al., 2020 [44]	Multiple countries	December 2019–March 2020	95	95	NGS/WGS	Whole genome
33	Laamarti et al., 2020 [45]	Morocco	NR	6	6	Oxford NanoporeTechnologies [ONT]	ORF1ab, S gene, 5′UTR
34	Leung et al., 2021 [46]	Hong Kong	as of February 2020	50	50	Nanopore and NGS	ORF3a, ORF1ab, S gene
35	Ling et al., 2020 [47]	Sweden	February–May 2020	348	348	NGS	5′-UTR, ORF1ab, S, ORF3a, M and N gene
36	McNamara et al., 2020 [48]	USA	March–May 2020	175	175	NGS	S and 3′UTR
37	Micheli et al., 2020 [49]	Italy	February–April 2020	20	20	NGS	M and N gene
38	Nagy et al. 2021 [50]	Worldwide	December 2019–September 2020	149,061	149,061	NGS	ORF1ab, ORF3a, ORF8, ORF6, N and S gene
39	Pachetti et al., 2020 [51]	Worldwide	December 2019–March 2020	220	215	NGS/WGS	Whole genome
40	Parvez et al., 2021 [52]	Bangladesh	as of August 2020	311	311	NGS/WGS	ORF1a, S and N gene
41	Raghav et al., 2020 [53]	India	March–June 2020	202	202	NGS	ORF1ab, 5′-UTR, ORF3a, ORF6, ORF7b, ORF84, ORF10, M, N and S
42	Rito et al., 2020 [54]	Worldwide	May–20	26,869	20,163	NGS/WGS	Whole genome
43	Saha et al., 2020 [55]	India	NR	566	566	NGS	5′UTR, ORF1ab, ORF3a, S, M and N
44	Saha et al., 2020 [56]	Bangladesh	April–July 2020	41	41	NGS	ORF1ab, ORF3a, ORF6, ORF7a, ORF8, Matrix (M gene), S and N gene
45	San et al., 2021 [57]	South Africa	March–June 2020	109	109	NGS	ORF1ab, ORF3a, ORF6, ORF7a, ORF7b, ORF8, ORF10, S, E, M and N gene
46	Skums et al., 2020 [58]	Worldwide	NR	319	274	NGS/WGS	Whole genome
47	Soliman et al., 2021 [59]	Egypt	June 2020	1	1	NGS	ORF1ab and S gene
48	Soratto et al., 2020 [2]	Sweden	April 2020	4	4	NGS	ORF1ab, ORF3a, ORF7a, S and N gene
49	Sun et al., 2020 [60]	China	NR	1	1	RT-PCR	E gene
50	Surleac et al., 2020 [61]	Romania	January–February 2020	25	25	NGS	ORF1ab, S and N gene
51	Taboada et al., 2020 [62]	Mexico	February–March 2020	17	17	NGS	ORF1ab, ORF8 and S gene
52	Toyoshima et al., 2020 [63]	Multiple countries	As of May 2020	12,343	12,343	NGS	ORF1ab, ORF3a, ORF8, S, N and M gene
53	Velasco et al., 2020 [64]	The Phillipines	April–July 2020	23	23	NGS	ORF1ab, ORF6, ORF7a, OORF7b, ORF8, ORF10, S, N and M gene
54	Volz et al., 2021 [65]	UK	January–June 2020	26,986	21,231	NGS	S gene
55	Wang et al., 2020 [66]	USA	July 2020	24,715	24,715	NGS	ORF1ab, ORF3a, ORF8 and S gene
56	Wang et al., 2020 [67]	Multiple countries	as of October 2020	75,775	75,775	NGS	ORF1ab
57	Wang et al., 2020 [68]	Worldwide	as of June 2020	15,140	15,140	NGS	Whole genome
58	Yap et al., 2020 [69]	Multiple countries	January–April 2020	142	112	NGS	ORF1ab, ORF8, S and N gene
59	Yuan et al., 2020 [70]	Worldwide	January–May 2020	11,183	11,183	NGS	Whole genome
60	Zhang et al., 2020 [71]	China	June–July 2020	6	6	NGS	ORF1ab gene, S and N gene
61	Ziegler et al., 2020 [72]	Germany	July 2020	1	1	qRT-PCR, PCR & Sanger sequencing	N gene
62	Zuckerman et al., 2020 [73]	Isreal	March 2020	8	8	qRT-PCR, NGS	5-UTR, ORF1ab, S, ORF3a and N gene

NGS: Next Generation Sequencing; WGS: Whole Genome Sequencing; RT-PCR: Reverse transcriptase PCR; qRT-PCR: Quantitative Reverse Transcription PCR; ORF: Open Reading Frame; S gene: Spike gene; N gene: Nucleocapsid phosphoprotein gene; M gene: Membrane glycoprotein gene; E gene: Envelope gene. (Additional information regarding the reported mutations and their types is provided in Supplementary Table S1). Sequences downloaded from databases such as NCBI and GSAID are presumed to be detected by NGS/WGS unless authors specify. ORF1ab includes Nsp1-14, RdRp, ORF1a, ORF1b, helicase, 3′ to 5′ exonuclease, endoRNAse, 2′-O-ribose methyltransferase.

**Table 2:** Subgroup analysis for comparison of SARS-CoV-2 variants across the country.

Country of Study	Number of Studies	Prevalence (%)	95% CI	*I*^2^ (%)	*Q*	Heterogeneity Test	
						DF	*p*
Bangladesh	3	98.7	91.9–99.8	57.445	4.700	2	0.095
China	3	97.5	85.1–99.6	5.356	2.113	2	0.348
Hong Kong	2	60.3	15.9–92.5	93.675	15.811	1	0.000
India	4	99.6	97.8–99.9	28.063	4.170	3	0.244
Israel	1	94.4	37.4–99.8	-	-	-	1.000
Italy	3	98.1	88.2–99.7	0.000	1.129	2	0.569
Korea	1	90.0	22.9–99.6	-	-	-	1.000
Mexico	1	97.2	56.0–99.9	-	-	-	1.000
Morocco	1	92.9	30.9–99.7	-	-	-	1.000
Multiple countries	6	98.2	95.3–99.3	95.168	103.483	5	0.000
Romania	1	98.1	65.2–99.9	-	-	-	1.000
Russia	1	99.8	94.3–100.0	-	-	-	1.000
Spain	2	99.6	96.3–100.0	73.466	3.769	1	0.052
Sweden	2	98.8	89.0–99.9	77.667	4.478	1	0.034
Taiwan	1	95.0	56.8–99.6	-	-	-	1.000
The Philippines	1	97.9	63.3–99.9	-	-	-	1.000
Turkey	1	99.2	82.5–100.0	-	-	-	1.000
UK	1	78.7	78.2–79.2	-	-	-	1.000
Uruguay	1	98.9	76.7–100.0	-	-	-	1.000
USA	5	98.4	94.8–99.5	92.301	51.954	4	0.000
Worldwide	10	90.3	82.6–94.8	99.747	3553.894	9	0.000
Total	51	97.4	94.4–98.8	98.952	4772.621	50	0.000

## Data Availability

The datasets used and/or analyzed during the current study are included in the manuscript.

## Supplementary Materials

The following are available online at www.mdpi.com/article/10.3390/life11111224/s1, Table S1: Major characteristics of the included studies; Table S2: Search strategy in four electronic databases; Table S3: Quality of included studies by JBI critical appraisal checklist for studies reporting prevalence data.
