# Supplementary material for: A Global Mutational Profile of SARS-CoV-2: A Systematic Review and Meta-Analysis of 368,316 COVID-19 Patients
Source: Life (Basel). 2021 Nov 11;11(11):1224. doi: 10.3390/life11111224 (PMC8620851; doi:10.3390/life11111224)
Supplement: Supplementary file 1 [file life-11-01224-s001.zip › life-1428310-supplementary.pdf]

life

MDPI

Systematic Review

A Global Mutational Profile of SARS-CoV-2: A Systematic Review and Meta-analysis of 368,316 Covid-19 Patients

Wardah Yusof [1], Ahmad Adebayo Irekeola [1,2], Yusuf Wada [1,3], Engku Nur Syafirah E.A.R. [1], Naveed Ahmed [1], Nurfadhlina Musa [4], Muhammad Fazli Khalid [5], Zaidah Abdul Rahman [1,6], Rosline Hassan [7], Nik Yusnoraini Yusof [5] and Chan Yean Yean [1,5,*]

[1] Department of Medical Microbiology and Parasitology, School of Medical Sciences, Universiti Sains Malaysia, Kubang Kerian 16150, Kelantan, Malaysia; wardahyusof@usm.my (W.Y.); irekeola@student.usm.my (A.A.I.); wadayusuf@student.usm.my (Y.W.); engkunursyafirah@student.usm.my (E. N.S.E.A.R.); naveed.malik@student.usm.my (N.A.); drzaidah@usm.my (Z.A.R.)
[2] Microbiology Unit, Department of Biological Sciences, College of Natural and Applied Sciences, Summit University, Offa 250101, Kwara State, Nigeria
[3] Department of Zoology, Faculty of Life Sciences, Ahmadu Bello University, Zaria 810107, Kaduna State, Nigeria
[4] Human Genome Centre, School of Medical Sciences, Universiti Sains Malaysia, Kubang Kerian 16150, Kelantan, Malaysia; fadhlina@usm.my
[5] Institute for Research in Molecular Medicine (INFORMM), Universiti Sains Malaysia, Kubang Kerian 16150, Kelantan, Malaysia; nikyus@usm.my (N.Y.Y.); fazlikhalid@usm.my (M.F.K.)
[6] Hospital Universiti Sains Malaysia, Universiti Sains Malaysia, Kubang Kerian 16150, Kelantan, Malaysia
[7] Department of Hematology, School of Medical Sciences, Universiti Sains Malaysia, Kubang Kerian 16150, Kelantan, Malaysia; roslin@usm.my
* Correspondence: yychan@usm.my; +60129011066

Citation: Yusof, W.; Irekeola, A.A.; Wada, Y.; Syafirah E.A.R., E.N.; Ahmed, N.; Musa, N.; Khalid, M.F.; Rahman, Z.A.; Hassan, R.; Yusof, N.Y.; et al. A Global Mutational Profile of SARS-CoV-2: A Systematic Review and Meta-analysis of 368,316 Covid-19 Patients. *Life* **2021**, *11*, 1224. https://doi.org/10.3390/life11111224

Academic Editor: Patrick Mercié

Received: 12 October 2021
Accepted: 08 November 2021
Published: 11 November 2021

Publisher's Note: MDPI stays neutral with regard to jurisdictional claims in published maps and institutional affiliations.

Copyright: © 2021 by the authors. Licensee MDPI, Basel, Switzerland. This article is an open access article distributed under the terms and conditions of the Creative Commons Attribution (CC BY) license (http://creativecommons.org/licenses/by/4.0/).

Table S1. Major characteristics of the included studies.

No.	Study ID (Ref)	Clades/ Lineage/ Type/ Cluster	Mutation (Loci/Country)	Database Used (Data Downloaded/for Alignment Purposes)
1	Akter *et al.* 2020 [7]	GR clade	ORF1ab: G339S, I120F, P323L, V480I, Q172R; Nucleocapsid phosphoprotein: R203K, G204R; Spike protein: D614G,	GSAID and NCBI
2	Andrés *et al.* 2020 [8]	NR	Spike protein: 13S-17V, 108T-110L, 140F-145Y, 199G-201F, 242L-246R, 341V-343N, 374F-S-396T, 393T-394N, 428D-442D, 428D-442D, 482G-484E, 622V-A-624I, 630T-P-632T, 654E-660Y, 660Y-679N, 817F-823F, 1006T-1007Y, 1167G	NCBI
3	Badua *et al.* 2020 [9]	NR	ORF1ab: L3606F, P4715L, V378I, S2839S, P6810S; Nucleocapsid phosphoprotein: R203K, G204R; Spike protein: D614G; ORF8: L84S	NCBI, GISAID EpiCoVTM
4	Barret *et al.* 2020 [10]	NR	Spike protein: D614G; 5'UTR: C241T; ORF1ab: C3037T (F924F), C14408T (P4715L) G29540A (only found in NY at >95% present in samples)	GISAID
5	Bartolini *et al.* 2020 [11]	Clade G, B1	P4715L, S5398A, L5F, D164G, D3G, R203K, G204R, C241T, C3037T, C14408T	GSAID
6	Becerra-Flores 2020 [12]	NR	D164G<br>Percentage from 3/30/2020-4/6/2020: Belgium 88.89%, France 84.82%, China 0.89%, Germany 47.62%, Netherlands 57.30%, Brazil 71.43%, Canada 42.40%, Italy 89.66%, Australia 17.91%, Japan 20.62%, Spain 41.30%, US 27.42%	European CDC
7	Benvenuto *et al.* 2020 [13]	NR	Spike protein: D614G; Nucleocapsid phosphoprotein: R203K, G204R	GSAID
8	Chang *et al.* 2020 [14]	NR	Orf1ab: T8517C; ORF8: S84L	GenBank
9	Chen *et al.* 2020[15]	Clade S and L	A208A, G227G, S1612T, I2385I, D2693D, S2839S, H3233Y, Y3722C, S3884L, N5020N, Y38Y, Q173Q, L94I, P36L, L84S, F274F, P344S, S23F	GSAID
10	Cusi *et al.* 2020 [16]	B1.1 lineage	5 single nucleotide changes: nt241, nt3037, nt14408, nt19839, nt23403 and nt28881	NCBI
11	Demİr *et al.* 2020 [17]	NR	Silent mutation: 106 (F), L37F, 280 (L), 139 (L), 71 (Y), 9 (T), 320 (L), 294 (D), 66 (V), 1682 (S).<br>Missense mutation: P323L, D614G, Q57H, V198I, R27C, M33I, R203K, G204R, A194L, A1006V, G309C, S188P, S485L, V10A >40% have: 106 (L) (61%), P323L (61%), D614G (61%) and Q57H (40%)	GSAIDs, NCBI
12	Devendran *et al.* 2021[18]	NR	ORF1ab: I147V, I671T, P2079L, P2144S, P4715L, A4798V, T5538I; Spike protein: Y144del, Q271R, R407I, D614G, C1250F; ORF8: L84S; Nucleocapsid phosphoprotein: T393I	NCBI
13	Du *et al.* 2020 [19]	Cluster C1, C2, C3, C4, C5, C6, C7<br>Lineage A, B<br>Sub lineage: A.1, A.2, A.3, A.4, A.5, A.p7, B.1, B.2, B.3, B.4, B.5, B.6, B.7. B.9. B.p11, B.15	17 highest frequency: C241T, C1059T (T265I), C3037T, C8782T, G11083T (L3606F), C14408T (P4715L), C14805T, C17747T (P5828L), A17858G (Y5865C), C18060T, A23403G (D614G), G25563T (Q57H, G26144T, T28144C (L84S), G28881A (R203K), G28882A (R203K), G28883C (G204R)	GSAID
14	Elizondo *et al.* 2020 [20]	Clades S, V, G, GH, & GR.<br>S clade (March), G clades (G, GH, GR) (April-May)	Clade S: L84S, C8782T, C17470 T, C25521 T, and C26088 T<br>Clade G: D614G, C241 T, C3037 T, and C14408 T (P323L)	GSAID
15	Eskier *et al.* 2020[21]	NR	ORF1ab: C14408T (P323L) & A23403G (D614G)	GISAID
16	Gómez-Carballa *et al.* 2020[22]	Six lineages A2a4, A2a5, A2a10, B3a, and B9	A2a5: C241T–C3037T–C14408T–A20268G–A23403G; A2a4: C241T–C3037T–C14408T–A23403G plus characteristic MNP: GGG28881AAC; A2a10: C241T–C3037T–C14408T–A23403G–C29144T); B3a: C8782T–T9477A–C14805T–G25979T–T28144C–C28657T–C28863T; B9: C8782T–C26088T–T28144C. *accounted for 87.9% of all genomes in the Spanish database	GSAID
17	Gong *et al.* 2020[23]	Clade I, II, III, IV	First wave (from China) (Jan - Feb 2020)<br>ORF1ab: C8517T, K5526R, V378I, A4788G. C10809T, G21055A (G7019S), C19547T (S6248L); ORF8: 27848-28229del; ORF3a: G251V; Spike gene: H49Y, S884F<br><br>Second wave (from other countries) (March)<br>ORF1ab: C2772T, C14144T (P4715L); Spike gene: D614G; Nucleocapsid phosphoprotein: R203K, G204R	GSAID

18	Gupta 2020[24]	NR	By loci:<br>ORF1ab - D58E, L952P, E955K, S1498F, N1559T, A3203V, G4227R, A4297G, F4304L, P4715L, Y232C, F1657L, A1906V, V1973L, G2374R; Spike protein - Y145del, N354D, D364Y, R416I, S438F, Y508H, D614GG, Q675H, T791I, F797C, A930V, I1216T, P1263L; ORF3a - A31T, Q57H, V88L, H93Y, G196V, G251VV; Membrane glycoprotein - D3G, T175M. ORF7a - V74F, S81L. ORF8 - V62L, L84S; Nucleocapsid phosphoprotein - L121H, T148, S193I, S197L, R203K, G204R, G204R, I292T.<br><br>By country:<br>New Zealand - D58E, F1657L, V1973L, V62L, L84S, S81L<br>China - L952P, E955K, N354D, D364Y, I1216T, G251V, L84S, L121H, T148I<br>India - S1498F, P4715L, D614G, Y145del, R416I, S438F, A930V, V74F, R203K, G204R<br>Russia - N1559T, G4227R, P4715L, D614G, Q57H<br>USA - A3203V, Y232C, L84S<br>Mexico - A4297G, P4715L, D614G, R203K, G204R<br>Sweden - F4304L, F797C, G251V<br>Japan – P4715L, D614G<br>Australia - Y232C, G251V, L84S<br>Canada - A1906V<br>France - G2374R, Y508H, G251V<br>Scotland - P4715L, D614G, Q675H<br>Taiwan - T791I<br>England - P4715L, D614G, P1263L<br>Hungary - P4715L, D614G, A31T<br>Congo - P4715L, D614G, Q57H<br>Saudi Arabia - P4715L, D614G, Q57H<br>Cambodia - V88L<br>Wales - H93Y, S193I<br>Chile - G196V, L84S, S197L<br>Georgia - G196V, L84S, S197L<br>Finland - P4715L, D614G, D3G<br>Belgium - T175M<br>Brazil - P4715L, D614G, T175M, R203K, G204R<br>Kuwait - V74F; Spain - L84S, S197L<br>Switzerland - P4715L, D614G, R203K, G204R<br>South Korea - L84S<br>Denmark - P4715L, D614G<br>Germany - P4715L, D614G<br>Italy - P4715L, D614G<br>Luxembourg - P4715L, D614G<br>Netherlands - P4715L, D614G<br>Peru - P4715L, D614G, R203K, G204R<br>Panama - P4715L, D614G<br>Vietnam - P4715L, D614G, R203K, G204R<br>Nigeria - P4715L, D614G, R203K, G204R	GSAID
19	Hartley *et al.* 2021[25]	Clade 19A, 19B, 20A, 20C	Clade 19A: C8782T, Clade 19B: T28144C; Clade 20A (derivative of 19A): C3037T, C14408T and A23403G (D614G); Clade 20B: G28881A, G28882A and G28883C; Clade 20C: C1059T and G25563T.<br>Main mutation: ORF1ab: P323L/F, Spike gene: D614G	NCBI and GSAID
20	Hassan *et al.* 2020 [26]	NR	P4715L, P13L, Q57H, D614G, V5272I, Q677 H, T3058I, A892V, V6600A, M4588I, N3405H/I, L6082F, G5530C, L4721I, E156D, T2016K, L3606F, A4489V, L3338F, R203K, G204R, I671T, P2144S, A4798V, R408I, T827I, K5957R, T5538I, P2079L, L84S, L41F, D3681N, A930V, D1939G, S2242P, S1534I, S194L, K2029E, P7034L, C857T, V4181I,E583D, R78M, I35T, L54F, T572I, P302S, S202N, E110STOP, S32I, T5036M, K4081R, I121L, S2015R, N418N, E3962K, Q3390R, L204F, S171L, T3453A, D4776Y, G6039V, M5974I, M177I, L6909F, S31L, K1973R, M6723I, D4532G, K4451N, P5624L, P7034S, C1243F, W131C, D155Y, G120R, V62L, A152S, D348Y	NR
21	Yang *et al.* 2020[27]	Type I, Type II, Type III, Type IV, Type V, Type VI	ORF1ab: T265I, V378I, F924F, S2839S, L3606F, P4715L, Y4847Y, P5828L, Y5865C, L5932L; Spike protein: D614G; ORF3a: Q57H, G251V; ORF8: L84S; Nucleocapsid phosphoprotein: L139L, R203K, G204R	GISAID, NCBI GenBank and CNCB
22	Ip *et al.* 2020[28]	NR	W152L (G22017T)	NCBI and GSAID
23	Islam *et al.* 2020[29]	NR	Nucleocapsid phosphoprotein: R203K, G204R, P13L; ORF3a: Q57H; ORF8:L84S; ORF1ab: A97V, P323L, T1198K, L37F; Spike protein: D614G	GSAID

24	Jacob *et al.* 2020[30]	Lineage B.1/B.1.1/B.1.36, Lineage B.6,	D614G, R78M and L54F	GSAID
25	Jary *et al.* 2021[31]	NR	233 intraviral variants, only 4 the same: 3591T > C, Thr859Ile	NCBI
26	Jenjaroenpun *et al.* 2021[32]	Clade GH and GR	T265I, A3529V, P314L, D614G, G18C, Q57H, S201G, R203K, G204R	NCBI and GSAID
27	Khailany *et al.* 2020[33]	NR	By country:<br>China: L84S, T3090I, N2708S, F2908I, D7018N, I2244T, G2534V, K6958R, G2251S, D2579A, T3090I, P34S, N3833K<br>USA: L84S, S428N, V62L, Y5720C, D75E, P971L, S194L, V62L, L3606F<br>Brazil: G251V<br>Taiwan: L84S<br>Sweden: Y5720C<br>Italy: L3606F, G251V<br>Japan: L84S, A1176V, P344S, L140V, E3764D, P6083L, G32_L39del	NCBI, National Microbiology-Data Center (NMDC) and NGDC Genome Warehouse
28	Kim *et al.* 2020[34]	Major clades: S, V, and G. Subclades: G.1 and G.2	767 types of synonymous and 1,352 types of non-synonymous mutation.<br>High frequency: ORF8: L84S; Spike protein: D614G, ORF3a: G251V, Q57H; ORF1ab: P214L; Nucleocapsid phosphoprotein: R203K. G204R	GSAID
29	Kim *et al.* 2020[35]	NR	D614G, G504D, V524D, P579L	NR
30	Koyama *et al.* 2020[36]	Six major clades with basal D614G, L84S, L3606F, D448del and G392D.<br>14 subclades	Most common variants: 3037C > T, P4715L and D614G<br><br>Variants: F924F/F106F, P4715L/P323L, D614G, Q57H, T265I/T85I, L84S, 2839S/S76S, 203_204delinsKR, L5932L/L7L, Y5865C/Y541C, P5828L/P504L, L3606F/L37F, Y4847Y/Y455Y, G251V, L6668L/L216L, R5661R/R337R, P765S/P585S, N5020N/N628N, D448del/D268del, L6205L/L280L, I739V/I559V, T175M, S3884L/S25L, Y717Y/Y537Y, G392D/G212D, S24L 164, 36C > T Non-coding 5′-UTR, A876T/A58T, S194L, V378I/V198I, D128D, L139L, A6245V/A320V, P13L, S197L, F3071Y/F308Y, G196V, 29742G > T Non-coding 3′-UTR, V13L, N824N 29870C > A Non-coding 3′-UTR, V62L, A69A, 27_37del Non-coding deletion 5′-UTR, 19_24del Non-coding deletion 5′-UTR, H83H/H83H, S T723T, P971L/T1198K	Chinese National Microbiology Data Center, Chinese National Genomics Data Center Genome Warehouse, GISAID, NCBI
31	Kozlovskaya *et al.* 2020[37]	NR	From sample: F924, D1036E, P4715L, D614G, R203K, G204R, F3568<br><br>In 218 Russian GSAIDS sequence: D614G, G204R, L3606F, M5900I, P4715L, S6831R, T175M, R203K, Gln57H, L890F, M1312I, P309L, S202N, T265I, N140T, G3278S, L84S, M5557I, P959S, T4364I, A8S, G97S, P3315S, T4644N, A364S, G379E, P3395L, A152S, G4777S, D3G, G4949H, N140T, D1036E, D198G, H113Y, A23S, A2V	GSAID
32	Kumar *et al.* 2020[38]	NR	H49Y, Y28N, Deletion and frameshift of amino acid at position 145, S221W, F157L, G181V, S247R, H655Y, F797C, N824X, A930V	NCBI database
33	Laamarti *et al.* 2020[39]	Clade 2A	Synonymous: F924F and L4715L, nonsynonymous: D614G, intergenic: 241C>T	GSAID
34	Leung *et al.* 2021[40]	NR	ORF3a: G251V; ORF1ab: H3233Y; Spike protein: L8V	NCBI
35	Ling *et al.* 2020[41]	Clades O, L, V, S, GR, G, GH	Synonymous: T265I, F924F.<br>Non-synonymous: C241T, T4175T, A43V, P323L, V341I, D614G, D936Y, Q57H, T175M, R203K, G204R	GSAID
36	McNamara *et al.* 2020[42]	NR	57%: D614G + C214T/C3037T/C14408T	GSAID
37	Micheli *et al.* 2020[43]	Clade 20B	Membrane glycoprotein: D3G; Nucleocapsid phosphoprotein: R203K, G204R, V246I	GSAIDS for sequence alignment
38	Nagy *et al.* 2021[44]	NR	Mild outcome<br>ORF8: L84S; ORF1ab: L37F, F308Y; ORF3a: G196V; Nucleocapsid gene: S197L.<br><br>Severe outcome<br>Spike protein: L54F, D614G, V1176F, ORF1ab: S1197R, T1198K, L71F, A97V, P323L; ORF3a: Q57H, G251V, S253P. Nucleocapsid gene: P13L, S194L, R203K, G204R, I292T; ORF6: I33T.	GSAIDS
39	Pachetti *et al.* 2020[45]	NR	By continents:<br>Asian – ORF1ab: nt1397 (V198I), nt8782, nt11083, ORF3a: nt26143, ORF8a: nt28144.	GSAID

			Oceania - ORF1ab: nt1397 (V198I), nt8782, nt11083, ORF3a: nt26143, ORF8a: nt28144.<br>Europe - ORF1ab: nt2891, nt3036, nt8782, nt11083, nt14408, Spike protein: nt23403 (D614G), ORF3a: nt26143, ORF8a: nt28144, Nucleocapsid: nt28881 (R203K, G204R).<br>North America - ORF1ab: nt1397 (V198I), nt3036, nt8782, nt11083, nt14408, 17746, 17857, nt18060, ORF3a: nt26143, ORF8a: 28144, Nucleocapsid: nt28881 (R203K, G204R).<br><br>By period:<br>Dec 19th 2019: No mutation (Asian).<br>Jan 1st -15th 2020: Asian - nt8782, nt26143, nt28144. Europe: nt11083, nt26143.<br>Jan 16th -31st 2020: Asia - nt17746, nt8782, nt11083, nt26143, nt28144. Oceania - nt26143. Europe - nt3036, nt8782, nt11083, nt26143, nt28144. North America - nt8782, nt11083. nt28144.<br>Feb1st-15th20: Asia - nt26143. Europe - nt8782, nt11083, nt26143, nt28144.<br>Feb 16th -28th 2020: Asia - nt8782, nt11083, nt26143. Oceania- nt11083. Europe: nt2891, nt3036, nt1083, nt14408, nt23403, nt28881. North America - nt8782, nt17746, nt28881.<br>March 1st - 13th 2020: Asia - nt26143, Oceania - nt17743, nt11083. Europe - nt3036, nt8782, nt11083, nt14408, nt23403, nt26143, nt28881, nt28144. North America - nt8782, nt11083, nt14408, nt17746, nt17857, nt18060, nt23403, nt26143, nt28144	
40	Parvez *et al.* 2021[46]	NR	Missense mutations: I300F, N2155S, Q2702H, D3355G, K3353R, V3572M, P3952S, P214L, E1084D, V1883T, D623G, F1118L, Q38L, S40L, Q57H, G172C, W27L, A65V, L84S, S202N, R203K, G204R, K373N, D377G.	GSAID
41	Raghav *et al.* 2020[47]	NR	most common (>15%)<br>Spike protein: D614G; 5′UTR: 241 C > T; ORF1ab: P4715L, F942F; Nucleocapsid gene: 28881 G > A (R203K), 28882 G > A (R203R), 28883 G > C (G204R), ORF8: L84S	NCBI, GSAID
42	Rito *et al.* 2020[48]	Clade A: Subclades A1, A2, A3, A4, A6, A7, A8<br>Clade B: Subclades B1, B1, B2, B3, B3, B4	20,247 different positions of the genome	GSAID
43	Saha *et al.* 2020[49]	NR	10% population: C241T, C3037T, C6310A, C6312A, G11085T, G11085A, C13732T, C14410T, C18879T, C22446T, A23405G, C23931T, G25565T, C26737T, C28313T, C28856T, G28883A, G28884A, G28885C	GSAID
44	Saha *et al.* 2020[50]	Clade A2a and B4	I120F, M418V, P129L, G339S, V469A, M494I, I1559X, I1672S, N1337S, P968S, S1424F, L373M*,E595D, E177D, A465V, A1766V, L373M*, E595D, T64I, K1077N, Q172R, N51D, S688L*, P109L, C296F*, Q1884H, D92G, K90R, V3M, L37F, V120L, L22I, T93I, P10S, A27T, P323L,A550V*, A423V, E263D, V459I, S273I, M218I, D614G, F1109L, T95I, E516Q*, L518I*, S98F, D92G, H146Y, D138Y, L5F, G172C, P25S, S40L, Q57H, Q38L, K75N, V48F, G254stop, E194Q, A2V, H125Y, W27L, Q62E, A65V, L84S, R203K, G204R,L230F,D377G, S202N, S194L, K373N, D377G, Q9H<br>*Novel mutation	GSAID
45	San *et al.* 2021	NR	C241T, C3037T, C14408T, A23403G, A12240G, G18181T, A11556T, A13003G, A17929C, T17928G, T25312A, T1483C, T20135A	NR
46	Skums *et al.* 2020[52]	NR	NR	GSAID
47	Soliman *et al.* 2021[53]	NR	Spike protein: S813I, Q677H, D614G, S12F; ORF1ab: A859V	GSAID
48	Soratto *et al.* 2020[54]	Lineage and Clade: 20C/B.1/G and 20B/B.1.1/GR.	T265I, K798N, R1628C, T2007I, K2029N, L3606F, T4217I, P4715L, L5F, T547I, D614G, R634H, D936Y, Q57H, R203K, G204R, S206P, K338N	GSAID
49	Sun *et al.* 2020[55]	NR	12-bp deletion in the E gene at the position of 26320–26331	NR
50	Surleac *et al.* 2020[56]	Lineage B.1.5 and B.1.1	Spike protein: D614G; Nucleocapsid phosphoprotein: R203K, G204R; ORF1ab: C3037T, P323L, T19839C, T870I, Y397C, A22803C, C28603T, A20268G, K489E	GSAID, NCBI
51	Taboada *et al.* 2020[57]	Lineage B/S, Lineage A/G (which includes sub-lineage A2 and A2a)	C241T, D614G, H48Y, P314L, L84S, C18060T	GSAID, NCBI

52	Toyoshima *et al*. 2020[58]	NR	Most common - ORF1ab: T265I, V378I, Y5865C, P5828L, A4489V, T2016K, L3606F, P4715L; Spike protein: D614G; Nucleocapsid phosphoprotein: P13L, G204R, R203K; Membrane glycoprotein: T175M; ORF3a: G251V, Q57H; ORF8: L84S,<br><br>By countries<br>China: ORF1ab: V378I, L3606F, P4715L; ORF3a: G251V; ORF8: L84S; Spike protein: D614G.<br>Korea: ORF1ab: L3606F; ORF3a: G251V; Nucleocapsid phosphoprotein: P13L; ORF8: L84S.<br>Japan: ORF1ab: L3606F, P4715L; ORF3a: Q57H, Spike protein: D614G, Nucleocapsid phosphoprotein: G204R, R203K; ORF8: L84S.<br>India: ORF1ab: T265I, V378I, A4489V, T2016K, L3606F, P4715L; ORF3a: Q57H, Nucleocapsid phosphoprotein: G204R, R203K, P13L, Spike protein: D614G.<br>Singapore: ORF1ab: L3606F, P4715L, A4489V, T2016K, T265I, ORF3a: Q57H; Spike protein: D614G; Nucleocapsid phosphoprotein: G204R, R203K, P13L; Membrane glycoprotein: T175M, ORF3a: G251V; ORF8: L84S.<br>England: ORF1ab: Y5865C, P5828L, L3606F, P4715L; ORF3a: Q57H, G251V; Nucleocapsid phosphoprotein: G204R, R203K; Membrane glycoprotein: T175M, Spike: D614G<br>Iceland: ORF1ab: Y5865C, P5828L, T265I, L3606F, P4715L; Nucleocapsid phosphoprotein: G204R, R203K, Membrane glycoprotein: T175M, ORF3a: G251V, Q57H; ORF8: L84S, Spike protein: D614G<br>Netherlands: ORF1ab: T265I, L3606F, P4715L; ORF3a: Q57H, G251V; Nucleocapsid phosphoprotein: G204R, R203K, M: T175M, Spike protein: D614G<br>Greece: ORF1ab: P4715L, L3606F; Spike protein: D614G; ORF3a: Q57H, G251V; Nucleocapsid phosphoprotein: G204R, R203K, ORF8: L84S<br>Portugal: ORF1ab: P4715L, T265I, L3606F; ORF3a: Q57H, G251V; Nucleocapsid phosphoprotein: G204R, R203K; Membrane glycoprotein: T175M, ORF8: L84S; Spike protein: D614G<br>Brazil: ORF1ab: P4715L, T265I, A4489V, T2016K, L3606F; ORF3a: Q57H, G251V; Nucleocapsid phosphoprotein: G204R, R203K; Membrane glycoprotein: T175M, P13L; ORF8: L84S; Spike: D614G<br>Italy: ORF1ab: P4715L, L3606F, T265I; ORF3a: Q57H, G251V; Nucleocapsid phosphoprotein: G204R, R203K, Membrane glycoprotein: T175M, Spike protein: D614G<br>Switzerland: ORF1ab: L3606F, P4715L, T265I; Nucleocapsid phosphoprotein: G204R, R203K, ORF3a: Q57H, G251V, Spike protein: D614G<br>Belgium: ORF1ab: P4715L, L3606F, T265I; ORF3a: Q57H, G251V; Nucleocapsid phosphoprotein: G204R, R203K; Membrane glycoprotein: T175M; Spike protein: D614G<br>Hungary: ORF1ab: P4715L, T265I; ORF3a: Q57H; Nucleocapsid phosphoprotein: G204R, R203K; Spike protein: D614G<br>USA: ORF1ab: P4715L, L3606F, P5828L, Y5865C, T265I; ORF3a: Q57H, G251V; Nucleocapsid phosphoprotein: G204R, R203K; Membrane glycoprotein: T175M; ORF8: L84S; Spike protein: D614G<br>Taiwan: ORF3a_Q57H, ORF1ab_T265I, Nucleocapsid phosphoprotein: G204R, R203K, ORF1ab_V378I, ORF3a_G251V, ORF1ab_A4489V, ORF1ab_T2016K, N_P13L, ORF8_L84S, ORF1ab_L3606F, S_614G, ORF1ab_P4715L<br>Australia: ORF1ab: P4715L, L3606F, V378I, T265I, A4489V, P5828L, Y5865C, T2016K; ORF3a: Q57H, G251V, Nucleocapsid phosphoprotein: G204R, R203K, P13L; ORF8: L84S, Spike protein: D614G<br>Canada: ORF3a_Q57H, ORF1ab_T265I, Nucleocapsid phosphoprotein: G204R, N_R203K, ORF1ab_V378I, ORF1ab_Y5865C, ORF1ab_P5828L, ORF1ab_A4489V, ORF1ab_T2016K, N_P13L, ORF8_L84S, ORF1ab_L3606F, S_614G, ORF1ab_P4715L<br>Thailand: ORF1ab: P4715L, L3606F, V378I, T265I; ORF3a: Q57H; Nucleocapsid phosphoprotein: G204R, R203K, Membrane glycoprotein: T175M, ORF8: L84S; Spike protein: D614G<br>Spain: ORF1ab: P4715L, L3606F, V378I; ORF3a: G251V; Nucleocapsid phosphoprotein: G204R, R203K; Membrane glycoprotein: T175M, ORF8: L84S, Spike protein: D614G<br>Denmark: ORF1ab: P4715L, T265I; ORF3a: Q57H, Nucleocapsid phosphoprotein: G204R, R203K, Membrane glycoprotein: T175M, Spike protein: D614G<br>Congo: ORF1ab: P4715L, L3606F; ORF3a: Q57H, Nucleocapsid phosphoprotein: G204R, R203K, ORF8: L84S, Spike protein: D614G	GSAID

			Germany: ORF1ab: P4715L, L3606F, Y5865C, V378I, T265I; ORF3a: Q57H; Nucleocapsid phosphoprotein: G204R, R203K, Membrane glycoprotein: T175M, Spike protein: D614G<br>Sweden: ORF1ab: P4715L, L3606F, T265I; ORF3a: Q57H; Nucleocapsid phosphoprotein: G204R, R203K, Spike protein: D614G<br>Finland: ORF1ab: P4715L, L3606F, T265I; ORF3a: Q57H, G251V; Nucleocapsid phosphoprotein: G204R, R203K, Membrane glycoprotein: T175M; Spike protein: D614G<br>France: ORF1ab: P4715L, L3606F, T265I; ORF3a: Q57H, G251V; Nucleocapsid phosphoprotein: G204R, R203K, Spike protein: D614G<br>Luxemburg: ORF1ab: P4715L, L3606F, T265I, ORF3a: Q57H, G251V; ORF8: L84S, Nucleocapsid phosphoprotein: G204R, R203K, Spike protein: D614G	
53	Velasco *et al.* 2020[59]	Clade O: Lineage B.6.<br>Clade GR: Lineage B.1.1 and B.1.1.28	T2016K, L3606F, A88V, P13L, P314L, H1087Y, S12F, D614G, Q8P, G204R, G212V, T2154I, S316I, L29F, V33I, T791I, T2274I, A3070V, R226K, E1126K, L3930F, V1176F, A903V, A1049V, A2282V, A12G, S206Y, E1126K, R80T, V21I, V1294F, A1031E, S114N, R209I, V188TI, H110Y, V1271L, A1643V, M2259I, S3173G, S67F	GSAID
54	Volz *et al.* 2021[60]	Lineage B.1	D614G	Coronavirus Disease 2019 (COVID-19) Genomics UK Consortium (CoG-UK) (COG-UK, 2020),
55	Wang *et al.* 2020[61]	Cluster A, B, C and D	4968 single mutations are detected. 11 mutations in the has a frequency greater than 700:<br>Synonymous mutation: C3037T (F106F), C8782T (S76S), and C18060T (L7L)<br>Missense mutation: P323L, D614G, Q57H, T85I, L84S, Y541C, P504L, and S24L	GSAID
56	Wang *et al.* 2020[62]	NR	G11083T (L37F)<br>Singapore, Japan, Turkey, Jordan, India, Norway, Australia, South Korea, United Kingdom, Canada, Vietnam, Belgium, Malaysia, France, United States, Brazil, Spain, Russia	GSAID
57	Wang *et al.* 2020[63]	Clusters I-VI	Cluster I: C3037T, C14408T<br>Cluster II: C3037T, C14408T, A23403G<br>Cluster III: C14408T<br>Cluster IV: C3037CT, C14408T, A23403G, G28881A, G28882A, G28883C<br>Cluster V: C 3037T, C14408T, A23403G, G25563T<br>Cluster V: CI8782T, T28144C	GSAID
58	Yap *et al.* 2020[64]	Clade S, L, V, G	Cluster I: L84S (T28144C), C8782T, G24047A, G2013T, G4390T, C2942T, 383nt Deletion in ORF8 (in Singapore only)<br>Cluster II: D614G (A23403G), C241T, C3037T, C14408T, G25563T G28881A, G28882A, G28883A<br>Cluster III: C6312A, G11083T, C13730T, C19524T, C23929T, C28311T<br>G26144T (G251V)- Cluster unknown<br><br>By country and date:<br>Cambodia (27/1/20) - No mutation.<br>Malaysia (Date: 28/1-22/1/20): C28782T, T28144C; 18/3-20/3/20- C8782T, G11083T.<br>The Philippines (Date: 8/3-28/3/20): C8782T, G11083T.<br>Singapore (Date: 23/1-10/2/20): C8782T, T28144C, G11083T, G26144T, T28144C. (Date: 13/2-2/3/20): C8782T, 4/3-31/3/20 - C8782T, T28144C, C241T, A23403G, G11083T, G25563T, G26144T, T28144C, G28882A, C3037T; 7/4-15/4/20 - G11083T.<br>Thailand (Date: 8/1-28/3/20): 8782T, T28144C, C241T, A23403G, G11083T, G25563T, T28144C, G28882A, C3037T.<br>Vietnam (Date: 22/1-7/3/20): 8782T, T28144C, C241T, A23403G, T28144C, G28882A, C3037T	GSAID
59	Yuan *et al.* 2020[65]	Five major clades 19A, 19B, 20A, 20B and 20C	Top SNPs with major count: ORF1ab: C14408T (P4715L)(Clade 20A), C3037T (Clade 19A), C1059T (Clade 20C), Spike gene: A23403G (D614G) (Clade 19A); ORF3a: G25563T in (Clade 20C), Nucleocapsid gene: G28881A (R203K) (Clade 20B), G28883C (G204R) (Clade 20B), G28882A (R203K) (Clade 20B). C8782T (Clade 19B)	GSAID
60	Zhang *et al.* 2020[66]	L-lineage European branch I	C241T, C3037T, C14408T (P4715L), A23403G (D614G), G28881A (R203K), G28882A (R203K), G28883C (G204R), C6026T	NCBI
61	Ziegler *et al.* 2020[67]	NR	C29200T, C28858T, C29451T	NCBI and GSAID

62	Zuckerman *et al.* 2020[68]	Clade 20 = 20B and 20C	A23403G (D164G), C14408T, G25563T, C1059T, G28881A, G28882A, G28883C, G15243T, C241T, C3037T, C18877T, C16626T, A3651G, A5541C	NCBI

NCBI: National Center for Biotechnology Information (https://www.ncbi.nlm.nih.gov/); GSAID: GSAID Initiative (https://www.gisaid.org/).NR: Not reported.

Table S2. Search strategy in four electronic databases.

No	Database	Search Strategies
1	PubMed	“(((((((2019-nCoV[Title/Abstract]) OR (coronavirus disease 2019[Title/Abstract])) OR (coronavirus disease-19[Title/Abstract])) OR (novel coronavirus[Title/Abstract])) OR (COVID-19[Title/Abstract])) OR (SARS-CoV-2[Title/Abstract])) AND (((((mutation[Title/Abstract]) OR (mutational[Title/Abstract])) OR (mutated[Title/Abstract])) OR (mutating[Title/Abstract])) OR (mutant[Title/Abstract])))) AND ((((((((((superspreading[Title/Abstract]) OR (super-spreader[Title/Abstract])) OR (super spreader[Title/Abstract])) OR (super spreaders[Title/Abstract])) OR (transmissibility[Title/Abstract])) OR (transmission[Title/Abstract])) OR (viral load[Title/Abstract])) OR (symptoms[Title/Abstract])) OR (severe[Title/Abstract])) OR (mortality[Title/Abstract])) OR (fatality[Title/Abstract]))).”
2	Scopus	“TITLE-ABS (2019-nCoV OR coronavirus disease 2019 OR coronavirus disease-19 OR novel coronavirus OR COVID-19 OR SARS-CoV-2) AND TITLE-ABS (mutation OR mutational OR mutated OR mutating OR mutant) AND TITLE-ABS (superspreading OR super-spreader OR super spreader OR super spreaders OR transmissibility OR transmission OR viral load OR symptoms OR severe OR mortality OR fatality).”
3	Science Direct	“(2019-nCoV OR coronavirus disease 2019 OR coronavirus disease-19 OR novel coronavirus OR COVID-19 OR SARS-CoV-2) (mutation OR mutational OR mutated OR mutating OR mutant) (superspreading OR super-spreader OR super spreader OR super spreaders OR transmissibility OR transmission OR viral load OR symptoms OR severe OR mortality OR fatality.”
4	Google Scholar	1. “(2019-nCoV OR coronavirus disease 2019) (mutation OR mutational) (superspreading OR super-spreader OR super spreader OR super spreaders OR transmissibility)”* 2. “(coronavirus disease-19 OR novel coronavirus OR COVID-19 OR SARS-CoV-2) (mutated OR mutating OR mutant) (transmission OR viral load OR symptoms OR severe OR mortality OR fatality)”*

*abstract within a year

Table S3. Quality of included studies by JBI critical appraisal checklist for studies reporting prevalence data.

No.	Study Name	Check List									Overall
	Author	1	2	3	4	5	6	7	8	9	
1	Akter *et al.* 2020	YES	YES	YES	YES	YES	YES	YES	NA	YES	8
2	Andrés *et al.* 2020	YES	YES	YES	YES	YES	YES	YES	NA	YES	8
3	Badua *et al.* 2020	YES	YES	YES	YES	YES	YES	YES	NA	YES	8
4	Barret *et al.* 2020	YES	YES	YES	YES	YES	YES	YES	NA	YES	8
5	Bartolini *et al.* 2020	YES	YES	YES	YES	YES	YES	YES	NA	YES	8
6	Becerra-Flores 2020	NO	YES	NO	NO	YES	YES	YES	NA	YES	5
7	Benvenuto *et al.* 2020	YES	YES	YES	YES	YES	YES	YES	NA	YES	8
8	Chang *et al.* 2020	YES	YES	YES	YES	YES	YES	YES	NA	YES	8
9	Chen *et al.* 2020	YES	YES	YES	YES	YES	YES	YES	NA	YES	8
10	Cusi *et al.* 2020	YES	YES	YES	YES	YES	YES	YES	NA	YES	8
11	Demİr *et al.* 2020	YES	YES	YES	YES	YES	YES	YES	NA	YES	8
12	Devendran *et al.* 2021	YES	YES	YES	YES	YES	YES	YES	NA	YES	8
13	Du *et al.* 2020	YES	YES	YES	YES	YES	YES	YES	NA	YES	8
14	Elizondo *et al.* 2020	YES	YES	YES	YES	YES	YES	YES	NA	YES	8

15	Eskier *et al.* 2020	YES	YES	YES	YES	YES	YES	YES	NA	YES	8
16	Gómez-Carballa *et al.* 2020	YES	YES	YES	YES	YES	YES	YES	NA	YES	8
17	Gong *et al.* 2020	YES	YES	YES	YES	YES	YES	YES	NA	YES	8
18	Gupta 2020	YES	YES	YES	YES	YES	YES	YES	NA	YES	8
19	Hartley *et al.* 2021	YES	YES	YES	YES	YES	YES	YES	NA	YES	8
20	Hassan *et al.* 2020	YES	YES	YES	YES	YES	YES	YES	NA	YES	8
21	Yang *et al.* 2020	YES	YES	YES	YES	YES	YES	YES	NA	YES	8
22	Ip *et al.* 2020	YES	YES	YES	YES	YES	YES	YES	NA	YES	8
23	Islam *et al.* 2020	YES	YES	YES	YES	YES	YES	YES	NA	YES	8
24	Jacob *et al.* 2020	YES	YES	YES	NO	YES	YES	YES	NA	YES	7
25	Jary *et al.* 2021	YES	YES	YES	NO	YES	YES	YES	NA	YES	8
26	Jenjaroenpun *et al.* 2021	YES	YES	YES	NO	YES	YES	YES	NA	YES	8
27	Khailany *et al.* 2020	YES	YES	YES	NO	YES	YES	YES	NA	YES	8
28	Kim *et al.* 2020	YES	YES	YES	YES	YES	YES	YES	NA	YES	8
29	Kim *et al.* 2020	YES	YES	YES	YES	YES	YES	YES	NA	YES	8
30	Koyama *et al.* 2020	YES	YES	YES	YES	YES	YES	YES	NA	YES	8
31	Kozlovskaya *et al.* 2020	YES	YES	YES	YES	YES	YES	YES	NA	YES	8
32	Kumar *et al.* 2020	YES	YES	YES	YES	YES	YES	YES	NA	YES	8
33	Laamarti *et al.* 2020	YES	YES	YES	YES	YES	YES	YES	NA	YES	8
34	Leung *et al.* 2021	YES	YES	YES	YES	YES	YES	YES	NA	YES	8
35	Ling *et al.* 2020	YES	YES	YES	YES	YES	YES	YES	NA	YES	8
36	McNamara *et al.* 2020	YES	YES	YES	YES	YES	YES	YES	NA	YES	8
37	Micheli *et al.* 2020	YES	YES	YES	YES	YES	YES	YES	NA	YES	8
38	Nagy *et al.* 2021	YES	YES	YES	YES	YES	YES	YES	NA	YES	8
39	Pachetti *et al.* 2020	YES	YES	YES	YES	YES	YES	YES	NA	YES	8
40	Parvez *et al.* 2021	YES	YES	YES	YES	YES	YES	YES	NA	YES	8
41	Raghav *et al.* 2020	YES	YES	YES	YES	YES	YES	YES	NA	YES	8
42	Rito *et al.* 2020	YES	YES	YES	YES	YES	YES	YES	NA	YES	8
43	Saha *et al.* 2020	YES	YES	YES	YES	YES	YES	YES	NA	YES	8
44	Saha *et al.* 2020	YES	YES	YES	YES	YES	YES	YES	NA	YES	8
45	San *et al.* 2021	YES	YES	YES	NO	YES	YES	YES	NA	YES	7
46	Skums *et al.* 2020	YES	YES	YES	YES	NO	YES	YES	NA	YES	7
47	Soliman *et al.* 2021	YES	YES	YES	YES	YES	YES	YES	NA	YES	8
48	Soratto *et al.* 2020	YES	YES	YES	YES	YES	YES	YES	NA	YES	8
49	Sun *et al.* 2020	YES	YES	YES	NO	YES	YES	YES	NA	YES	7
50	Surleac *et al.* 2020	YES	YES	YES	YES	YES	YES	YES	NA	YES	8
51	Taboada *et al.* 2020	YES	YES	YES	YES	YES	YES	YES	NA	YES	8
52	Toyoshima *et al.* 2020	YES	YES	YES	YES	YES	YES	YES	NA	YES	8
53	Velasco *et al.* 2020	YES	YES	YES	YES	YES	YES	YES	NA	YES	8
54	Volz *et al.* 2021	YES	YES	YES	YES	YES	YES	YES	NA	YES	8
55	Wang *et al.* 2020	YES	YES	YES	YES	YES	YES	YES	NA	YES	8
56	Wang *et al.* 2020	YES	YES	YES	YES	YES	YES	YES	NA	YES	8
57	Wang *et al.* 2020	YES	YES	YES	YES	YES	YES	YES	NA	YES	8
58	Yap *et al.* 2020	YES	YES	YES	YES	YES	YES	YES	NA	YES	8
59	Yuan *et al.* 2020	YES	YES	YES	YES	YES	YES	YES	NA	YES	8
60	Zhang *et al.* 2020	YES	YES	YES	YES	YES	YES	YES	NA	YES	8
61	Ziegler *et al.* 2020	YES	YES	YES	YES	YES	YES	YES	NA	YES	8
62	Zuckerman *et al.* 2020	YES	YES	YES	YES	YES	YES	YES	NA	YES	8

-Checklist questions: 1) Was the sample frame appropriate to address the target population?; 2) Were study participants sampled in an appropriate way?; 3) Was the sample size adequate?; 4) Were the study subjects and the setting described in detail?; 5) Was the data analysis conducted with sufficient coverage of the identified sample?; 6) Were valid methods used for the identification of the condition?; 7) Was the condition measured in a standard, reliable way for all participants?; 8) Was there appropriate statistical analysis?; 9) Was the response rate adequate, and if not, was the low response rate managed appropriately?,

-Score: '1' for "yes", '0' for other parameters; Score '7' to '9' were of sufficient quality.
